# An update to database TraVA: organ-specific cold stress response in *Arabidopsis thaliana*

**DOI:** 10.1186/s12870-019-1636-y

**Published:** 2019-02-15

**Authors:** Anna V. Klepikova, Ivan V. Kulakovskiy, Artem S. Kasianov, Maria D. Logacheva, Aleksey A. Penin

**Affiliations:** 1 Institute for Information Transmission Problems of the Russian Academy of Sciences, Bolshoy Karetny per. 19, build.1, Moscow, 127051 Russia; 2 Vavilov Institute of General Genetics, Russian Academy of Sciences, Gubkina 3, Moscow, 119991 Russia; 3 Institute of Mathematical Problems of Biology RAS - the Branch of Keldysh Institute of Applied Mathematics of Russian Academy of Sciences, Vitkevicha 1, Pushchino, Moscow Region, 142290 Russia; 4 Engelhardt Institute of Molecular Biology, Russian Academy of Sciences, Vavilova 32, 119991 Moscow, Russia; 5 Moscow State University, Leninskye gory, build 1, Moscow, 119992 Russia; 6 Skolkovo Institute of Science and Technology, Nobelya Ulitsa 3, Moscow, 121205 Russia

**Keywords:** Cold stress, *Arabidopsis thaliana*, RNA-seq, Transcriptome map, Organ-specific stress response

## Abstract

**Background:**

Transcriptome map is a powerful tool for a variety of biological studies; transcriptome maps that include different organs, tissues, cells and stages of development are currently available for at least 30 plants. Some of them include samples treated by environmental or biotic stresses. However, most studies explore only limited set of organs and developmental stages (leaves or seedlings). In order to provide broader view of organ-specific strategies of cold stress response we studied expression changes that follow exposure to cold (+ 4 °C) in different aerial parts of plant: cotyledons, hypocotyl, leaves, young flowers, mature flowers and seeds using RNA-seq.

**Results:**

The results on differential expression in leaves are congruent with current knowledge on stress response pathways, in particular, the role of *CBF* genes. In other organs, both essence and dynamics of gene expression changes are different. We show the involvement of genes that are confined to narrow expression patterns in non-stress conditions into stress response. In particular, the genes that control cell wall modification in pollen, are activated in leaves. In seeds, predominant pattern is the change of lipid metabolism.

**Conclusions:**

Stress response is highly organ-specific; different pathways are involved in this process in each type of organs. The results were integrated with previously published transcriptome map of *Arabidopsis thaliana* and used for an update of a public database TraVa: http://travadb.org/browse/Species=AthStress.

**Electronic supplementary material:**

The online version of this article (10.1186/s12870-019-1636-y) contains supplementary material, which is available to authorized users.

## Background

Since the construction of the first plant transcriptome map [1], gene expression atlases were published for many plants belonging to the variety of families and became a widely used tool in plant studies. By definition, transcriptome map, or gene expression atlas, is a collection of expression profiles of all genes in different organs, tissues or cells under various environmental conditions [1]. For the current moment, such collections are covering plant taxa from moss [2] and pine [3] to many species of Rosids and Asterids, including model [4, 5], agricultural [6, 7] and ornamental species [8].

In the last decade transcriptome maps were applied in the various studies, including gene regulatory network inference, evolutionary analysis and dissection of the certain developmental processes [3, 9, 10]. However, despite of gene expression atlas definition, only few papers includes transcriptome profiles of samples under stress conditions as a part of main dataset [8, 11, 12] or for the verification of map completeness [5].

The process of increasing tolerance to freezing – cold acclimation – is well-studied in *Arabidopsis thaliana* [13]. The main regulator genes in *A. thaliana* were discovered in cold response studies using classic genetics methods. The CBF pathway plays a major role in cold acclimation [14]. *CBF* (*CBF1*, *CBF2*, and *CBF3*, also known as *DREB1b*, *DREB1c*, and *DREB1a*, respectively) genes are induced within 15 min of cold exposure [15–18]. These genes encode the AP2/ERF transcription factor (TF) family, which recognizes the C-repeat (CRT)/dehydration-responsive element (DRE) DNA regulatory element in the promoters of targeted genes [17, 19]. Under constitutive overexpression of *CBF* genes, a number of genes, which are referred as CBF regulons, are activated; these genes partly overlap a set of cold-regulated genes that have been identified by transcriptome studies [16, 17, 20, 21]. Beyond the CBF cold resistance pathway, other TFs also participate in cold acclimation. In particular, *ZAT12* expression increases in parallel with *CBF*s, and its overexpression results in a high freezing tolerance [22].

During the past 20 years, a great deal of effort has been made to fully characterize transcriptomic changes after low temperature treatment in *A. thaliana* [13]. Thousands of genes have been identified as cold-induced in microarray studies, this allowed a more complete understanding of the regulatory cascades controlling acclimation processes [22–25]. These works investigated various aspects of cold tolerance. For example, the paper by Kilian et al. [24] provides a detailed description of gene expression profiles in a time-course experiment. A work by Vogel et al. [22], analyzed transgenic lines with constitutive overexpression of TFs. Finally, Rasmussen et al. [26] explored variations in the cold response of 10 *A. thaliana* ecotypes. These studies highlight the considerable differences in the cold stress response regulatory networks for various ecotypes.

However, most papers focused on transcriptomic studies have been conducted on whole aerial parts of plants [23, 24] or leaves [25, 26]. To our knowledge, there have been no attempts to analyze and compare the expression profiles in different parts of plants during cold exposure. Also there is a lack of studies integrating stress data with developmental transcriptome maps. In this paper, we aimed to explore this question and analyzed expression in six parts of Arabidopsis plants after 3 and 27 h of cold treatment using RNA-seq. We identified differentially expressed genes between the stress and control samples and compared them between plant organs. This comparison revealed noticeable differences in the number and composition of DE genes and affected biological processes. We also observed differences in the behavior of CBF and other known cold-response genes between samples and found unique transcriptome features of these genes in Arabidopsis genes expression atlas. The data on expression profiles were uploaded in database TraVa (travadb.org) [5] and integrated with detailed transcriptome map of *A. thaliana*.

## Results and discussion

### Cold-related GO enrichment in transcriptome map under normal conditions

During the analysis of RNA-Seq based *Arabidopsis thaliana* transcriptome map [5] we detected differentially expressed (DE) genes between all pairs of samples to characterize general variability of gene expression profiles. For each comparison we calculated GO enrichment and found overrepresentation of GO terms GO:0009631~cold acclimation or GO:0009409~response to cold in 598 out of 3081 comparisons. In many cases such enrichment appeared between different parts of the same organ, as for petiole and lamina of young leaf, so the origin of the effect is unlikely due to perturbation during plant growing.

We have introduced a metric “DE Score” in *A. thaliana* transcriptome map, which corresponds to the number of pairwise comparisons where gene was DE and reflects both width of gene expression pattern and difference in the expression level. Genes that are annotated with the GO:0009631 and GO:0009409 categories have median DE Score 1326. This result indicates the involvement of the genes described as stress-response in various processes under normal conditions and the expression of these genes on different levels in different organs of plant.

Thus, the differential involvement of cold-associated genes in other biological processes rises a question about the variance of involvement in cold acclimation of these genes in different organs of plant.

### Choice of samples and sequencing

Since our study was aimed at analysis and comparison of stress responses in different parts of plants, we selected the most different biological samples using a recently constructed transcriptome map of *A. thaliana* [5]. Based on the clustering of samples, we chose the cotyledons and hypocotyl of seedlings, mature leaves, first flowers at the anthesis stage, flowers at stage 9 [27] and green mature seeds (samples are described in Additional file 1: Table S1). From another point of view, we focused on plants growing under conditions close to natural; thus, plants were cultivated in soil under a long day (16 h light/8 h dark)-light cycle, in contrast to many previous studies that used constant (24 h) light. The circadian rhythm is known to have great influence on the expression of stress-response genes [28, 29]; thus, each sample was collected at the same time during the light cycle. For each sample, 20–25 million reads were obtained. The mean Pearson r^2^ correlation between the replicates was 0.97 (Additional file 1: Table S2). Seeds, flowers and young flowers were clustered by biological samples, whereas leaves, cotyledons and hypocotyls were very close to one another and partly grouped by the time of stress treatment (Additional file 2). This is presumably caused by that all these organs are photosynthetic and thus have highly similar expression profiles and similar dynamics of response to stress.

### The number of differentially expressed genes varies greatly between samples

To investigate the differences in response to cold between organs, we identified DE genes between 3 h of cold treatment and the control and 27 h of cold treatment and the control for each sample (referred to hereafter as “Sample name 3” and “Sample name 27” after 3 and 27 h of cold treatment for a specific organ, respectively). We observed a notable difference in the number of up- and downregulated DE genes (from 1022 in Flower 3 to 6867 in Leaf 27). The ratio of down- to upregulated DE genes varied for different organs at the same time (0.57 in Leaf 3 and 1.32 in Hypocotyl 3) and for one organ at two time points (0.52 in Flower 3 and 1.4 in Flower 27) (Table 1).

**Table 1:** Number of differentially expressed genes

Sample	3 h of cold treatment		27 h of cold treatment	
	Downregulated	Upregulated	Downregulated	Upregulated
Cotyledons	855	1286	2885	3092
Hypocotyl	1238	937	3546	2699
Leaf	765	1351	3580	3287
Flower	352	670	1350	961
Young flower	603	560	2388	2134
Seeds	1112	1142	1766	1327

Overall, 15,459 genes were DE in at least one of the 12 cases (Additional file 1: Table S3). All of the consequent analyses were conducted on these 15,459 stress-response genes only.

### CBF regulon genes are activated under stress conditions

As mentioned above, earlier large-scale studies on the cold response focused on leaves or whole seedlings. Our results show high congruence with the results from these studies when considering leaves, cotyledons and the hypocotyl. In particular, Park et al. [21] dissected the CBF regulons by performing expression analysis of transgenic lines with overexpressed *CBF* genes under a constitutive cauliflower mosaic virus (CaMV) 35S promoter. For the Leaf, Cotyledons and Hypocotyl, the majority of DE genes matched the direction of the expression change in the transgenic lines and under cold treatment in our dataset (the percentage of matches varied from 50% in Hypocotyl 3 to 82% in Leaf 27) (Additional file 1: Tables S4 and S5). The lowest match percentage was in Seeds 3 (32%). This shows that our analysis provided consistent results for previously explored systems and has the potential to provide novel insights into organ-specific patterns in the stress response.

### The genes that are common to for all organs are stress-related

**Fig. 1:**
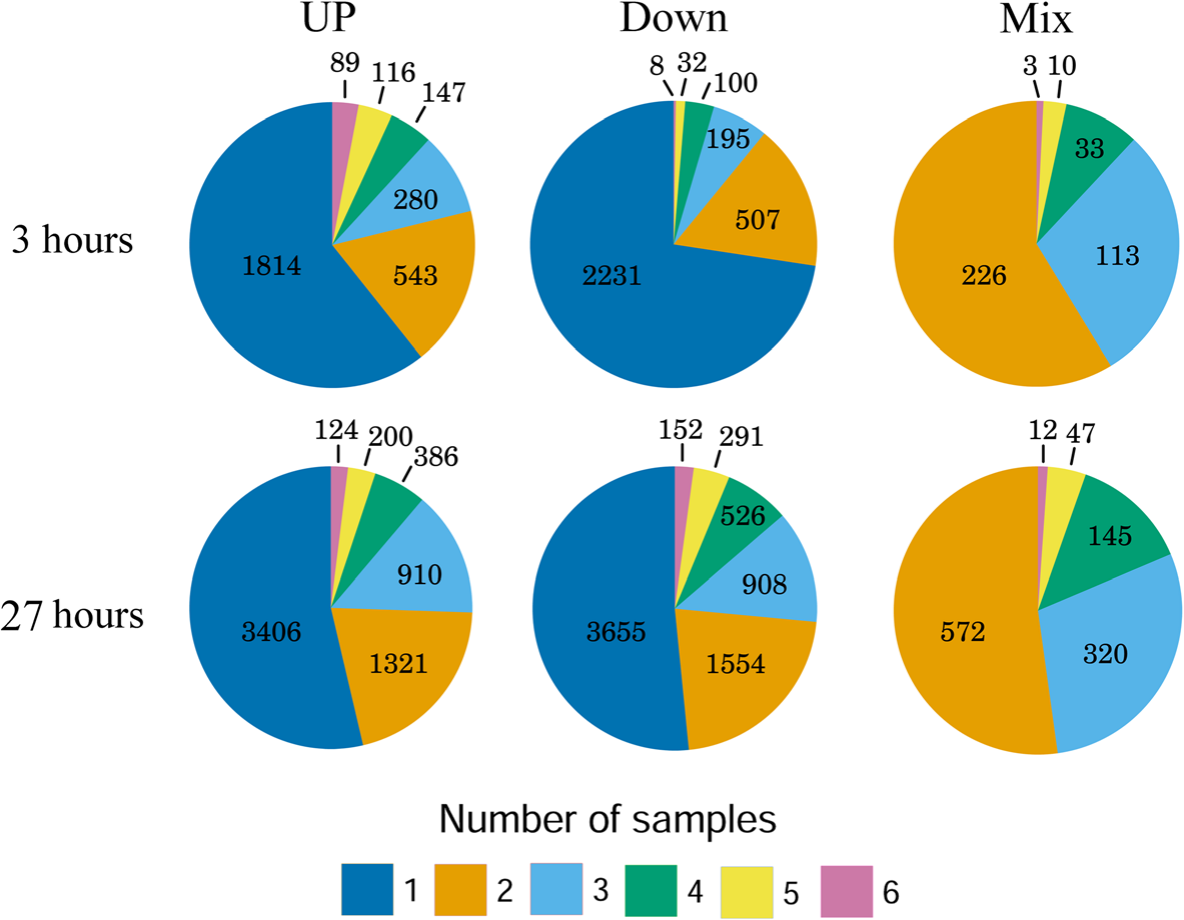
Number of differentially expressed genes in different numbers of samples. Down, Up and Mixed DE gene lists for each sample (3 and 27 h of cold treatment) were intersected, and genes unique for individual samples and common in 2, 3, 4, 5 or 6 samples were counted

We classified DE genes into the following three categories: genes that are downregulated in at least one sample and are not upregulated in any sample (“Down”), genes that are upregulated in at least one sample and are not downregulated in any sample (“Up”) and genes that are downregulated in at least one sample and upregulated in at least one sample (“Mix”). At 3 h, 3073 DE genes belonged to the Down category, 2989 to the Up category and only 385 of DE genes belonged to the Mix category. At 27 h, 7086 genes were in the Down category, 6347 in the Up category and surprisingly high number of DE genes, 1096, belong to the Mix category, revealing the differences in the involvement of genes in the cold response in different organs (Additional file 1: Table S6). We found an extremely low number of genes that were common for all organs in all of the categories. At 3 h, only 8 common genes were downregulated, 89 genes were upregulated at in all organs and 3 genes showed mixed pattern (i.e. were upregulated in several samples and downregulated in the others). After 27 h, the number of common genes increased to 152, 154 and 12 for the Up, Down and Mix categories, respectively (Fig. 1, Additional file 1: Table S7 and Additional file 3). Common DE genes in the Up category for both 3 and 27 h of cold treatment were strongly enriched in stress-related Gene Ontology (GO) categories (such as GO:0009631~cold acclimation) (Additional file 1: Table S8 and Additional file 4). The small amount of genes that were common for organs and their notable GO enrichment identified these genes as the most general and “core” genes for cold resistance. The increase in the number of common genes at 27 h showed the considerable specificity of the early response to stress in plant organs, which becomes more generalized during late stages of stress reactions.

The number of downregulated genes common for all or any 5 out of 6 samples at 3 h of cold treatment is very small and they do not have any significant GO enrichment. Genes downregulated after 27 h of cold treatment were enriched for photosynthetic terms, which was consistent with the inhibition of photosynthesis by low temperatures [30] (Additional file 1: Table S8 and Additional file 4).

### Genes unique for samples are involved in organ-specific processes

The number of genes that change the expression level in response to cold in an organ-specific manner varied from 90 for upregulated genes in Flower 3 and Young Flower 3 to 1115 for downregulated genes in Leaf 27 (Additional file 1: Table S7 and Additional file 3). In total, 4045 genes were organ-specific after 3 h of cold treatment, while 9780 genes were organ-specific after 27 h of cold treatment.

While common genes had stress-related enrichment, organ-specific DE genes were enriched in specific manner (Additional file 1: Tables S9 and S10). Downregulated genes in Cotyledons 3 were enriched in pentatricopeptide repeat (PPR) motif-containing genes, which have been shown to play an important role in organelle biogenesis and RNA editing [31, 32]. The same category was overrepresented in upregulated genes in Cotyledons 27. Among these genes, the PPR protein is encoded by the *SOAR1* gene, which has been shown to regulate cold, drought and salt stress responses [33]. After 27 h of exposure to cold, the downregulated genes in Leaf were enriched for photosynthesis. For the downregulated genes in Seeds 3 and 27, categories related to RNA splicing, response to fungus and lipid transport were enriched, while upregulated genes in Seeds 3 and 27 were overrepresented in lipid storage terms. Changes in the cell lipid composition leading to membrane stabilization are known to play a role in freezing resistance mechanisms [34] and may be of particular importance because the accumulation of lipids in developing seeds is a crucial process for plant reproduction [35]. Hypocotyl 27 showed photosynthetic enrichment in upregulated genes. Upregulated genes in Young Flower 27 were enriched for catabolic processes and pollen-related terms (Additional file 1: Tables S9 and S10).

The remarkable diversity of the processes leading to cold acclimation in various organs provides evidence for the adjustment of the general response to stress by organ-specific responses. This fact may limit the application of knowledge on the cold response in one organ (e.g. leaf) to another organ (e.g. seed).

### Stress response in non-leaf organs does not involve many known regulators and stress-response genes

**Fig. 2:**
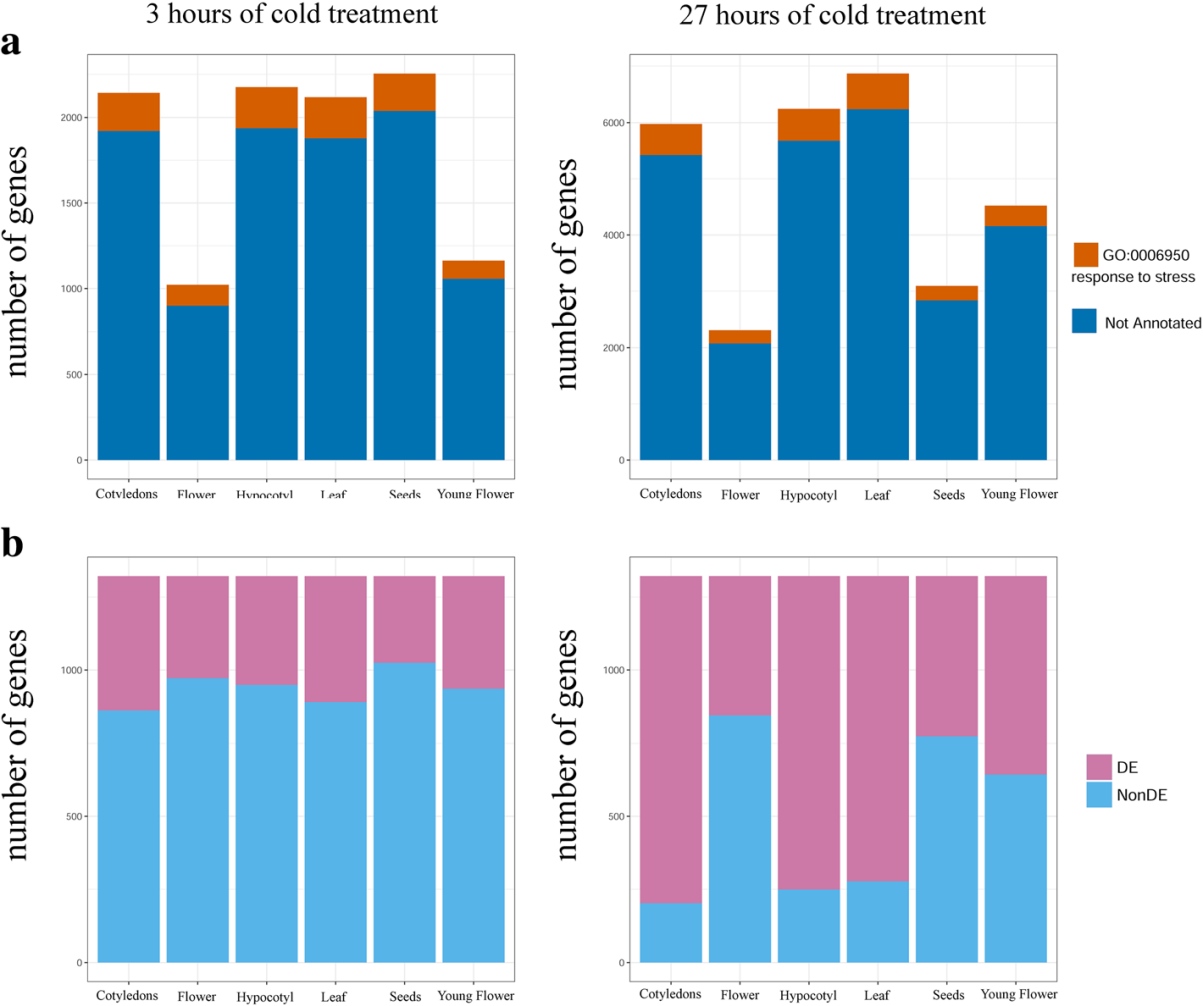
(**a**) Number of DE genes for each sample that are annotated or not annotated as stress-responses via Gene Ontology. (**b**) Number of COR genes that are DE in our data

We found a surprisingly low fraction of DE genes annotated as stress responsive (GO category “GO:0006950~response to stress” and downstream categories). The fraction of genes from this category varied between 8 and 12% for both 3 h and 27 h samples; the absolute number of stress-annotated genes was the lowest in the Young Flower 3 (106 genes) and highest in Leaf 27 (628 genes). These results show that GO annotation of the *Arabidopsis* genome strongly underestimates the number of stress-responsive genes. This is especially pronounced in organs that are not usually the focus of stress response studies, such as flowers and seeds (Fig. 2a). GO annotation has several shortcomings and is known to be incomplete; in particular, approximately 50% of *A. thaliana* genes do not have biological process annotations. Additionally, many GO annotations are based only on computational predictions and are not supported by experimental data [36].

Searching a list of genes for which participation in the cold stress response is defined by experimental data (1322 COR genes) [21], we found that 64% of these genes were DE in at least one organ after 3 h of cold treatment (26% belong to Down, 34% to Up, and 4% to Mix category) (Additional file 1: Table S11). Considering the genes by organ, the percentage of DE genes varied from 22% in Seeds 3 to 35% in Cotyledons 3 (Fig. 2b). After 27 h, the picture was even more pronounced. Although 99% of COR genes were DE in at least one organ, the distribution of genes by organ varied greatly; 79–85% of COR genes were DE in Cotyledons 27, Leaf 27, and Hypocotyl 27, although the percentages in Flower 27, Young Flower 27, and Seeds 27 were 36, 51 and 41%, respectively (Fig. 2b, Additional file 1: Table S12). These results show that the cold stress response in non-photosynthetic organs not only involves additional genes that were not previously associated with stress but also does not recruit many known regulators.

The *CBF1*, *CBF2* and *CBF3* genes are known to be activated within a few minutes after exposure to cold [15, 18]. Despite their crucial importance in cold acclimation, these genes are not unique in the early response to stress. Using the time course experiments performed by Kilian et al. [24], Park et al. [21], identified 27 transcriptional factors as having the same behavior as CBF-encoded genes (named “first-wave” genes). In our data, the first expression measurement time was 3 h after the beginning of the low temperature conditions, which is not the earliest possible time point, although we were still able to identify all except one of the first-wave genes as DE in at least one organ. Four genes were DE in all organs after 3 h of cold treatment (including *CBF3*), while others had noticeable differences in their expression patterns (Additional file 1: Table S13). For 5 genes, we observed opposite changes in expression in various organs, with high differences in fold changes (e.g., the fold change for *ZAT12* in Hypocotyl 3 and Leaf 3 were 0.22 and 2.96, respectively). After 27 h, the diversity of the expression changes became more notable; the first-wave genes from most organs showed downregulated expression or their expression had returned to the control value. However, in Leaf 27, there were 19 genes that were still upregulated (Additional file 1: Table S14). This variety in responses to cold from early activated transcriptional factors confirms the inadequacy of simple transfer or the results obtained on one organ to another.

### Expression characteristics of stress-response genes: Shannon entropy

**Fig. 3:**
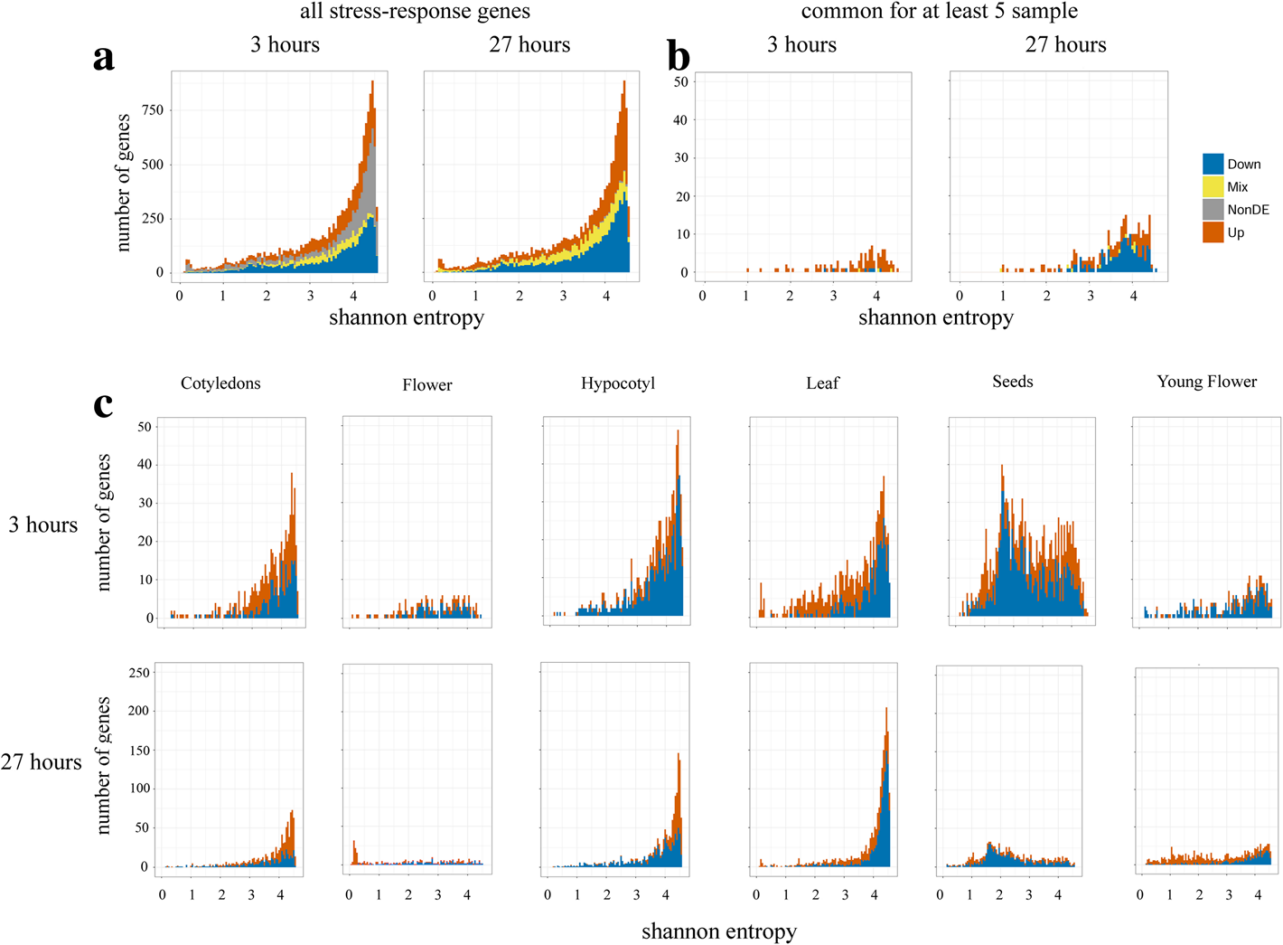
Shannon entropy distribution. (**a**) Shannon entropy for the 15,459 stress-response genes in the transcriptome map. (**b**) Shannon entropy for genes that are DE in at least 5 samples. (**c**) Shannon entropy for unique sample genes

We assessed several parameters that are associated with organ-specific stress-response genes based on the RNA-seq transcriptome map for *A. thaliana* [5]*.* We first estimated the expression pattern width using Shannon entropy H [37, 38]. Genes with high H value are expressed ubiquitously, while those with a low H value have a narrow expression pattern. The distribution of entropy for all of the expressed genes was noticeably skewed to the right, indicating a high number of genes with wide expression patterns [5]. The second small peak appears at very low H values and corresponds to genes that are highly expressed in an organ-, tissue- or stage-specific manner. The distribution for the 15,459 genes that have expression changes in response to stress resembled the overall distribution, while genes that are common in at least 5 organs genes lacked low H peaks (Fig. 3 a and b). The entropy of unique genes for most of the samples was distributed similarly to common DE genes. However, some samples had distinct features in the H distribution. Specifically, for genes that were upregulated in Leaf 3, there was a peak at low (0–0.3) entropy values (Fig. 3c). We analyzed the expression patterns of these genes in the transcriptome map. Surprisingly, under non-stress conditions, the expression of all of these genes (with one exception) as restricted to mature anthers and whole flowers containing anthers at the same stage (Additional file 1: Table S15). These genes were differentially expressed in Leaf after 3 h of cold treatment, and the half of them (44%) were also DEs in Leaf 27. Only 33% of the genes were DE in Flower 27, and none of them had shifted expression in Flower 3. For most of these genes, their function is related to controlling cell wall conditions and pollen tube growth (Additional file 1: Table S15). Among them, the most pronounced changes were in genes encoding pectin methylesterases. Pectin is a crucial component in the cell wall, as the matrix in which other polysaccharides (cellulose and hemicellulose) are embedded. Pectins are produced in the Golgi in a highly methylesterified form and are then modified by pectin methylesterases, which catalyze deesterification [39]. The ratio of esterified to de-esterified pectins determines many cell wall properties, such as rigidity, permeability and cohesion. This increase in pectin methylesterase activity under cold stress has been found in other plants [40, 41]. It is regarded as a cold acclimation mechanism because the increase in cell wall rigidity offers a higher resistance to dehydration and inhibits organ growth. Our results indicate that implementation of this mechanism in *A. thaliana* occurs by the recruitment of pollen-specific genes.

Similar to Leaf 3, the H distribution for DE genes uniquely upregulated in Flower 27 has a peak at low values (Fig. 3c). All of these genes are also anther-specific. GO enrichment analysis revealed overrepresentation of terms associated with pollen tube growth and cell wall modification (Additional file 1: Table S16). These genes revealed a complex picture. In particular, we observed concerted upregulation of pectin methylesterase inhibitors (*PMEI5* (*AT2G31430*) and others), while the expression of pectin methylesterases (*PPME1*, *VGD1*, *VGDH2* (*AT1G69940*, *AT2G47040*, and *AT3G62170*) was also increased. Pectin methylesterase PPME1 has been shown to linearly demethylesterify pectin chains in pollen tube walls. A reduction in PPME1 activity in ppme1 mutant leads to decreased cell wall rigidity [42]. *VGD1* is another gene that encodes a pectin methylesterase. VGD1 also has a linear demethylesterification activity and modifies pollen wall pectin [43]. Similar to PPME1, loss of VGD1 function results in a reduction in pollen tube wall strength. Both of these genes were 2–3-fold upregulated in Flower 27. We found a 2-fold increase in *CWINV2* expression (Additional file 1: Table S16). *CWINV2* is a member of the cell wall invertase gene family (CWINs), which encodes enzymes that degrade sucrose into glucose and fructose. CWIN activity is critical for anther development and the production of viable pollen [44]. In many cold-susceptible plants, cold stress reduces pollen sterility [45, 46]. Comparison of rice cultivars with contrasting reactions to cold showed that pollen-specific *CWIN* is downregulated in cold-susceptible cultivars and unchanged in cold-tolerant cultivars [46]. The increase in *CWINV2* is therefore presumably a mechanism in the cold acclimation pathway and is usually coupled with an increase in monosaccharide transporter activity, thus supplying sugars to the anthers [46]. However, we did not observe increased expression of *STP* genes, which encode monosaccharide transporters in *Arabidopsis thaliana*. We suggest that the gene expression landscape that we observe in Flower 27 is not a steady state, but could be reversed either to normal development if returned to non-stress conditions or to further unfolding due to cold acclimation reactions.

As mentioned above, we also observed genes that are involved in pollen tube growth. Polar growth is an essential property of normal pollen tube development and requires spatiotemporal regulation of F-actin arrangement. A family of Rho GTPases of plants (ROPs) participates in a range of biological processes, including pollen tube and root hair tip growth, hormone responses and biotic stress responses [47]. ROPs are under the control of positive regulator guanine nucleotide exchange factors (GEFs) [48]. For the family of ROPGEFs (GEFs containing plant-specific ROP nucleotide exchanger domain), involvement in pollen tube growth has been shown [49, 50]. *ROP1* is known to activate two competing F-actin assembly pathways that are controlled by *RIC4*, and F-actin disassembly is promoted by *RIC3* [51]. F-actin assembly leads to the accumulation of vesicles at the cell tip, whereas F-actin disassembly is required for exocytosis, and a proper balance of the two processes is necessary for polar growth [52]. In Flower 27, *ROPGEF8 (AT3G24620)* and *RIC3 (AT1G04450)* are two-fold upregulated (Additional file 1: Table S16). Additionally, the expression of two profilin genes – *PRF4* and *PRF5* – is two-fold increased. Profilin is a regulator of actin polymerization at the apical membrane in the pollen tube; *prf5* and *prf4* mutants have a decreased rate of pollen tube growth. The Flower sample corresponds to flower at anthesis, where pollination has already occurred. The increase in expression of genes responsible for pollen tube growth may indicate intensification of the fertilization rate.

In Seeds 3, the distribution of Shannon entropy is bimodal for both up- and downregulated genes (Fig. 3c). Seeds 27 lacks genes with a high H, and the medium H gene list strongly overlaps with genes in Seeds 3. Analysis of the expression patterns of genes with a medium H in the transcriptome map shows their specificity for seeds. The majority of genes in both cases are downregulated and enriched for F-box, plant defense GO and other category terms (Additional file 1: Table S17). Plant F-box is a large protein family, including 897 members in *A. thaliana*, and most of these genes are poorly studied [53]. *SON1* (*AT2G17310*) is an F-box domain-containing gene and it encodes component of systemic acquired resistance from pathogens [54]. *SCRL5 (AT1G60987)*, *LCR73 (AT2G02147)*, *RALFL3 (AT1G23147)* and other genes are referred to as small cysteine-rich antimicrobial peptides, including defensins, which are common for eukaryotes [55]. This class of genes is a known part of defense against pathogens [56]. In Seeds 3 (and, in most cases, Seeds 27), these genes are downregulated (Additional file 1: Table S17). Additionally, as was mentioned above, upregulated genes in Seeds 3 and 27 are enriched for lipid metabolism and storage.

### Expression characteristics of stress-response genes: The DE score

**Fig. 4:**
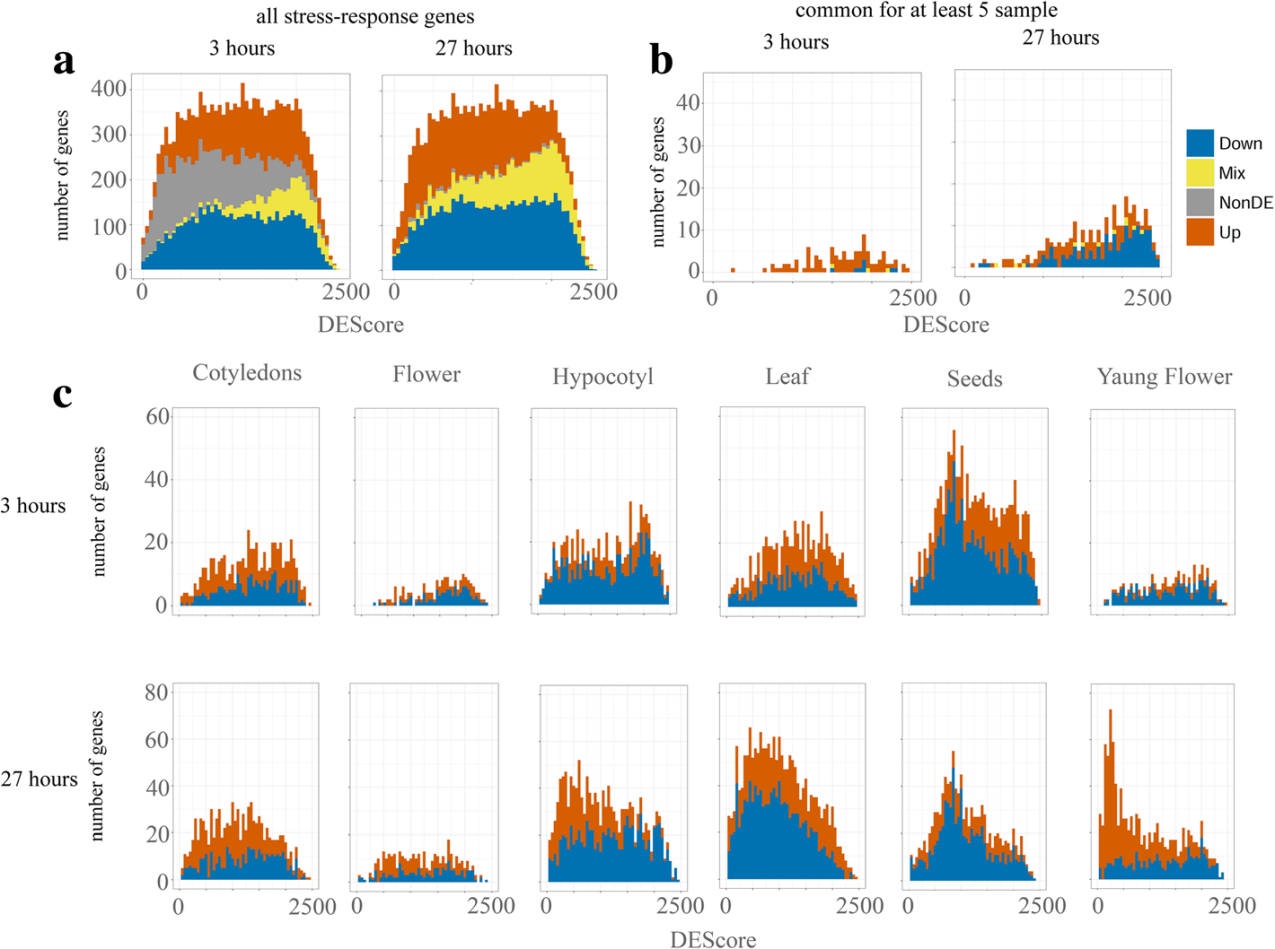
DE Score distribution. (**a**) DE Score for the 15,459 stress-response genes in the transcriptome map. (**b**) DE Score for genes that are DE in at least 5 samples. (c) DE Score for unique sample genes

Another parameter of gene expression is the DE Score. In Klepikova et al. [5], the differential expression between all pairs of transcriptome map samples was analyzed; the DE Score is the number of paired comparisons in which the gene is DE. The maximum possible DE Score is 3081 from 79 samples in the transcriptome map. We analyzed the distribution of the DE Scores for the genes whose expression changed under stress (Fig. 4). Genes with a low DE Score were underrepresented in the DE Score distribution for stress data compared with the distribution for all expressed genes (Fig. 4a). Genes from Mix 3 and 27 were skewed to a high DE Scores. DE Score reflects the variations of the levels of gene expression as well as the width of the expression pattern. A high DE Score indicates that a gene is expressed in multiple tissues and has significantly different expression levels in these tissues. Thus, genes that have opposite changes in expression in the cold response (Mix genes) may be involved in various processes in distinct tissues.

DE genes common in at least 5 organs had a DE Score distribution skewed to the right (Fig. 4b). The Shannon entropy H indicates a wide pattern of expression for common cold-response genes and supports the idea of the differential involvement of these genes in stress response n different organs.

The DE Score distributions of DE genes unique for organs were slightly skewed to the left (Fig. 4c). Most organs had no noticeable peaks in the distribution, whereas Young Flower 27 had a pronounced spike of mostly upregulated and some downregulated DE genes with a DE Score of less than 500 (Fig. 4c). According to the transcriptome map, the majority of these genes are specific to young anthers (from flower at stage 9 according to Smyth et al. [27]) and young flowers at similar stages. These genes have strong GO and other category enrichment, with terms associated with the ubiquitin-dependent protein catabolic process, regulation of transcription and F-box (Additional file 1: Table S18).

Ubiquitination is a key post-translational modification regulatory mechanism that is essential for plant development. The ubiquitin 26S proteasome system is important for protein modulation and removing defective proteins. For some genes that encode components of the ubiquitin 26S proteasome system, involvement in the abiotic stress response was observed [57].

Many F-box protein family members are subunits in the SCF (SKP1, CULLIN, FBX and RBX1) complex, which includes types of E3 ubiquitin ligases [58]. In total, 37 genes annotated as F-box domain-encoded were 2- to 13-fold upregulated in Young Flower 27 (Additional file 1: Table S18). For some Arabidopsis SKP-LIKE (ASK) proteins, another participant in the SCF complex, direct interaction with F-box proteins was shown [59]. In Young Flower 27, 8 *ASK* genes were significantly upregulated.

In a recent paper by Gladman et al. [60], two proteins from the NO APICAL MERISTEM/ARABIDOPSIS TRANSCRIPTION ACTIVATION FACTOR-1/CUP-SHAPED COTYLEDONS-2 (NAC) family, NAC78 and NAC56 were identified as positive regulators of ubiquitin 26S proteasome system genes. Though we did not observe expression changes in these genes in Young Flower 27, the DE gene list includes several other *NAC* genes, such as *NAC023* or *NAC063*, which could possibly be involved in the cold stress response in developing flowers.

### Genes unique to organs: Overrepresentation of regulatory elements from transcription factors outside the ERF/AP2 family

For a deeper understanding of the gene networks involved in cold acclimation in different organs, we analyzed the overrepresentation of regulatory elements from transcription factors from different gene lists (e.g., common in all or at least five organs, DE genes unique for certain samples, or genes with a certain Shannon entropy H; for a full list of the tested gene groups see Additional file 1: Table S19).

As expected, the promoter regions of DE genes upregulated in all or at least 5 organs were enriched with CBF regulatory elements. Genes which have these elements and are thus likely to be under regulation of CBF1–3 transcription factors displayed stress GO enrichment terms (Additional file 1: Table S20). As expected, promoter regions of DE genes upregulated in all or at least 5 organs were enriched with CBF regulatory elements. Genes that cause this overrepresentation and can be under regulation of CBF1–3 transcription factors have stress GO enrichment. Among these genes 12 transcription factors that also are characterized by overrepresentation in DE genes common for at least 5 organs and possibly regulated by them genes are enriched with stress GO terms too. Four of these transcription factors belong to ERF/AP2 family and were described as participants in both biotic and abiotic stress response [61].

Regarding genes that are unique for each sample, we did not find any overrepresentation of CBF regulatory elements, which shows that factors other than CBF govern organ-specific stress responses. In particular, we found an overrepresentation of regulatory elements for 9 NAC transcription factors in promoters of upregulated DE genes in Young Flower 27 (Additional file 1: Table S21).

### Database

**Fig. 5:**
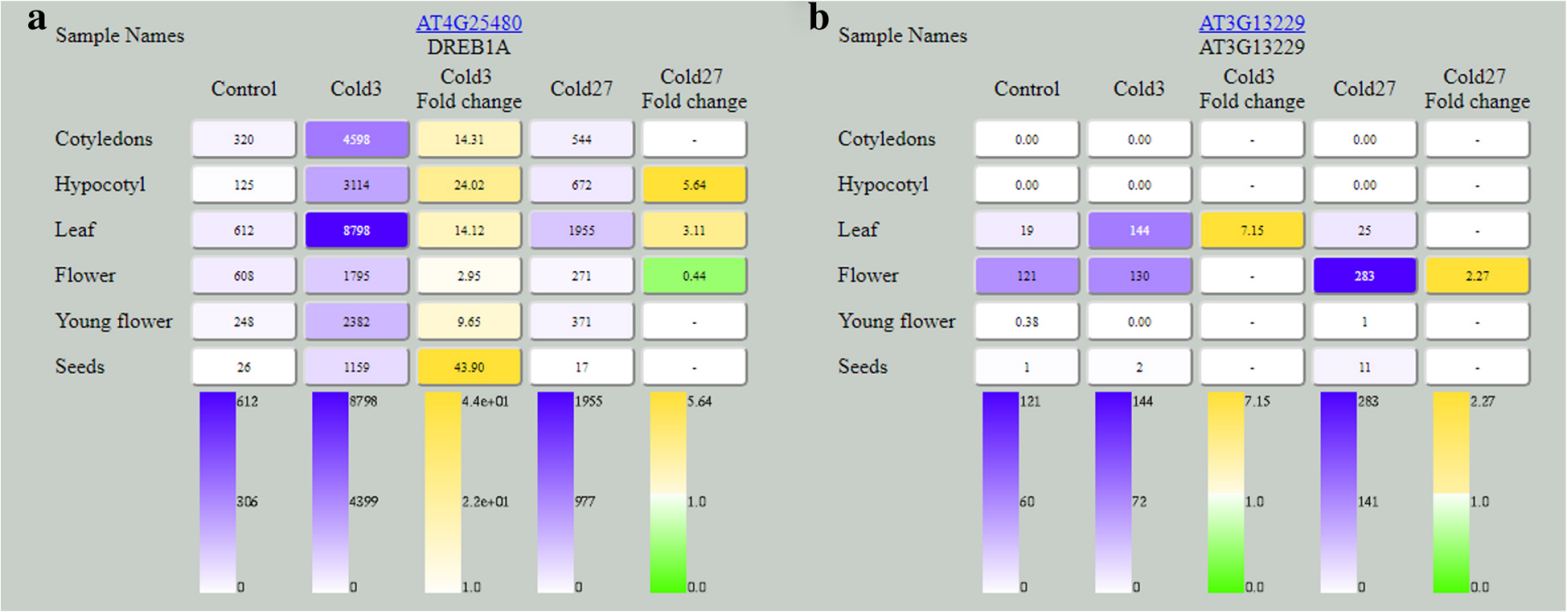
Database view. (**a**) Read counts and fold changes for *CBF3* (*DREB1A*) among all samples. (**b**) Read counts and fold changes for *AT3G13229* among all samples

To make these data available to the plant science community, we included them in our database TraVA (https://travadb.org). The interface and options are similar to the datasets from Klepikova et al. [5] and Kasianov et al. [10]. The profiles for each gene in the cotyledons, hypocotyl, leaves, young flowers, mature flowers and seeds are represented under both the control and cold treatment conditions as the number of reads and as fold change in the expression level relative to the control (Fig. 5).

## Conclusions

We analyzed gene expression in six Arabidopsis organs and tissues after 3 and 27 h of cold treatment using RNA-seq. We found that 15,459 genes were differentially expressed in at least one sample. Well-studied organs (leaf, cotyledons and the hypocotyl) showed similar results to other studies, while seeds, flowers and young flowers displayed pronounced differences. Only a small number of genes were common in all samples. These core genes were strongly enriched in stress-related GO categories. Unique sample genes were related to different processes in each organ. Some of these genes displayed expression specificities, such as peaks in Shannon entropy or DE Score distributions. Thus, while the mechanisms of cold stress response are common in all plants, in every organ they are modified in a unique fashion, including the recruitment of genes that are expressed in other organs in non-stress conditions.

## Methods

### Plant growth, cold treatment and sample collection

Col-0 *A. thaliana* (accession CS70000) plants were grown as described in Klepikova et al. [62], with the exception of vernalization. The collected samples are described in Additional file 1: Table S1. For each sample, two biological replicates with 15 individual plants were obtained and fixed in RNAlater (Qiagen, USA).

Control samples were harvested from ZT 8 to 9 and on the next day at ZT 5 temperature in a climate chamber set at + 4 °C. Samples treated with cold for 3 h were collected at ZT 8 and for 27 h at ZT 8 the next day.

### RNA extraction and sequencing

Total RNA was extracted with a RNeasy Plant Kit (Qiagen, USA) following the manufacturer’s protocol. cDNA libraries for sequencing were constructed with the TruSeq RNA Sample Prep Kits v2 (Illumina) following the manufacturer’s protocol. An Illumina HiSeq2000 was used for sequencing with a 50 bp read length and a sequence depth of 20 million uniquely mapped reads.

### Trimming and mapping of reads and expression level determination

For read trimming, the CLC Genomics Workbench 6.5.1 was used with the following parameters: “quality scores - 0.005; trim ambiguous nucleotides – 2; remove 5’-terminal nucleotides – 1; remove 3’-terminal nucleotides – 1; and discard reads below a length of 25”. The trimmed reads were mapped using the CLC Genomics Workbench to the reference *A. thaliana* genome (TAIR10 genome release) with unique mapping only (length fraction = 1 and similarity fraction = 0.95). For each gene, total gene reads (TGR) was determined as the sum of all the reads mapped on this gene. Sequencing and mapping statistics are shown at Additional file 1: Table S22. Total gene reads and RPKM are provided for all samples at Additional file 1: Table S23 and S24, respectively.

### Identification of differentially expressed genes

Differentially expressed (DE) genes were identified using the R package “DESeq2” [63]. A false discovery rate (FDR) of 0.05 and fold change of 2 were chosen as the initial threshold for significant differential expression.

### Gene ontology enrichment analysis

Downregulated and upregulated DE gene lists were analyzed by Gene Ontology (GO) and other annotation (as key words or as a protein domain) enrichments using the DAVID gene functional annotation tool with an FDR value of 0.05 and fold change category representation of 2 as the threshold of significance [64, 65].

### Hierarchical clustering

A hierarchical tree was obtained with the “hclust” function from the R package “stats” [66].

### Identification of key transcription factors

To identify the transcription factors involved in the regulation of observed differential gene expression we used annotations of the transcription factor targets based on ampDAP-seq [67]. For each set of DE genes, we considered data for all transcription factors. We estimated the relative enrichment of targets among differentially expressed genes as the log2 of the %target (DE) / %target (non-DE). The statistical significance was assessed using the right-tailed Fisher’s exact test with 2 × 2 contingency tables (targets vs. non-targets and DE vs. non-DE) with FDR correction for multiple tested transcription factors (219 TFs).

### Accession numbers

The Illumina sequence reads have been deposited into the NCBI Sequence Read Archive with project ID PRJNA411947.

## Additional files

Additional file 1: The description of the supplementary tables is located in the beginning of the xlsx file. (XLSX 15389 kb)

Additional file 2: Hierarchical clustering of samples (PDF 8 kb)

Additional file 3: Venn diagram of sample-specific DE genes (PDF 443 kb)

Additional file 4: Histogram of fold enrichment in groups of GO terms enriched in common for all or at least five samples genes (PDF 7 kb)

## Abbreviations

COR: Cold-response
DE: Differentially expressed
GO: Gene ontology
PPR: Pentatricopeptide repeat
TF: Transcription factor
