# Supplementary figures and images for: An update to database TraVA: organ-specific cold stress response in Arabidopsis thaliana
Source: BMC Plant Biol. 2019 Feb 15;19(Suppl 1):49. doi: 10.1186/s12870-019-1636-y (PMC6393959; doi:10.1186/s12870-019-1636-y)

# Hierarchical Clustering of Samples

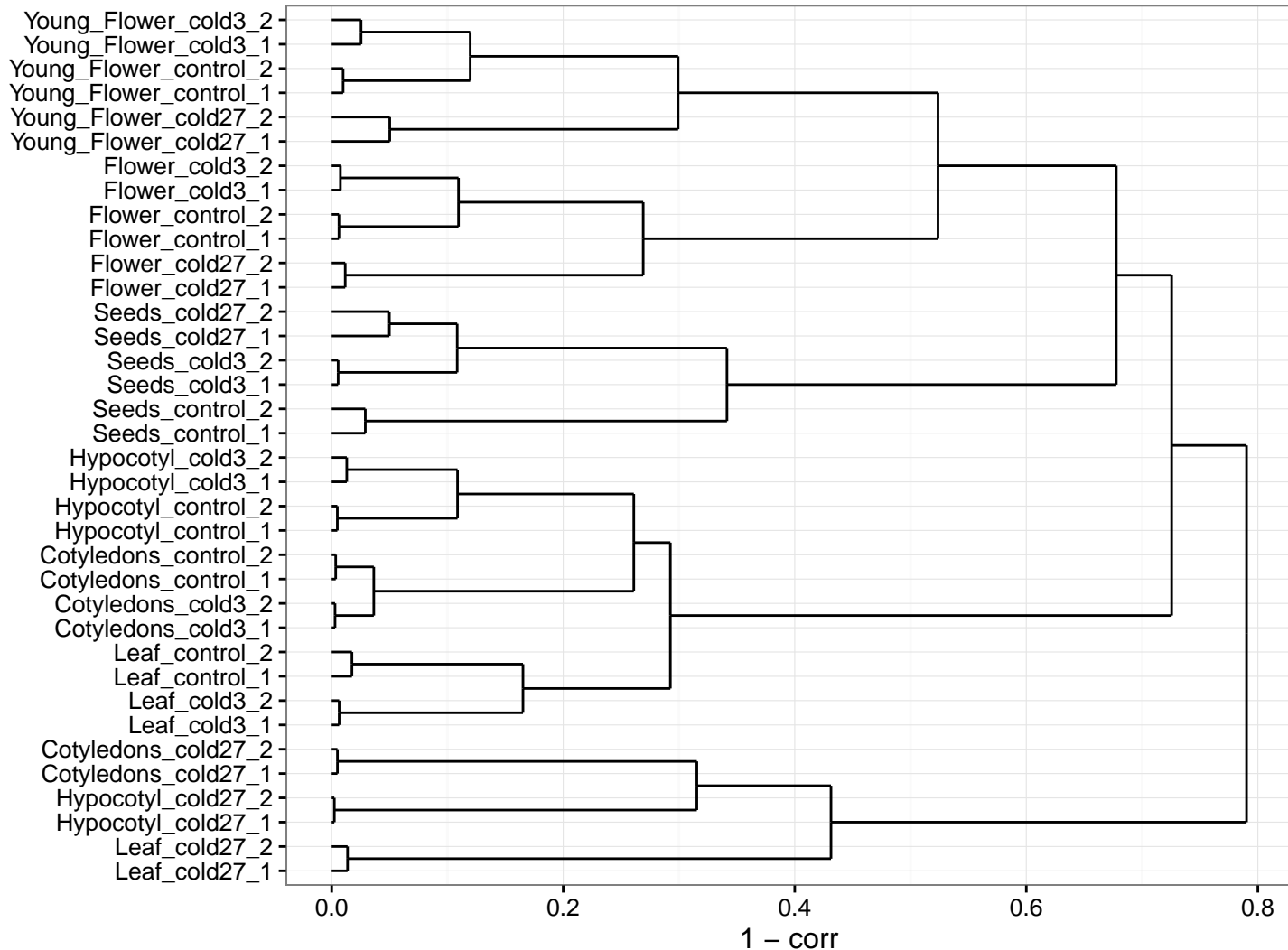

Supplement: Supplementary file 2 — Hierarchical clustering of samples (PDF 8 kb) [file 12870_2019_1636_MOESM2_ESM.pdf]
