# Supplementary material for: An update to database TraVA: organ-specific cold stress response in Arabidopsis thaliana
Source: BMC Plant Biol. 2019 Feb 15;19(Suppl 1):49. doi: 10.1186/s12870-019-1636-y (PMC6393959; doi:10.1186/s12870-019-1636-y)
Supplement: Supplementary file 3 — Venn diagram of sample-specific DE genes (PDF 443 kb) [file 12870_2019_1636_MOESM3_ESM.pdf]

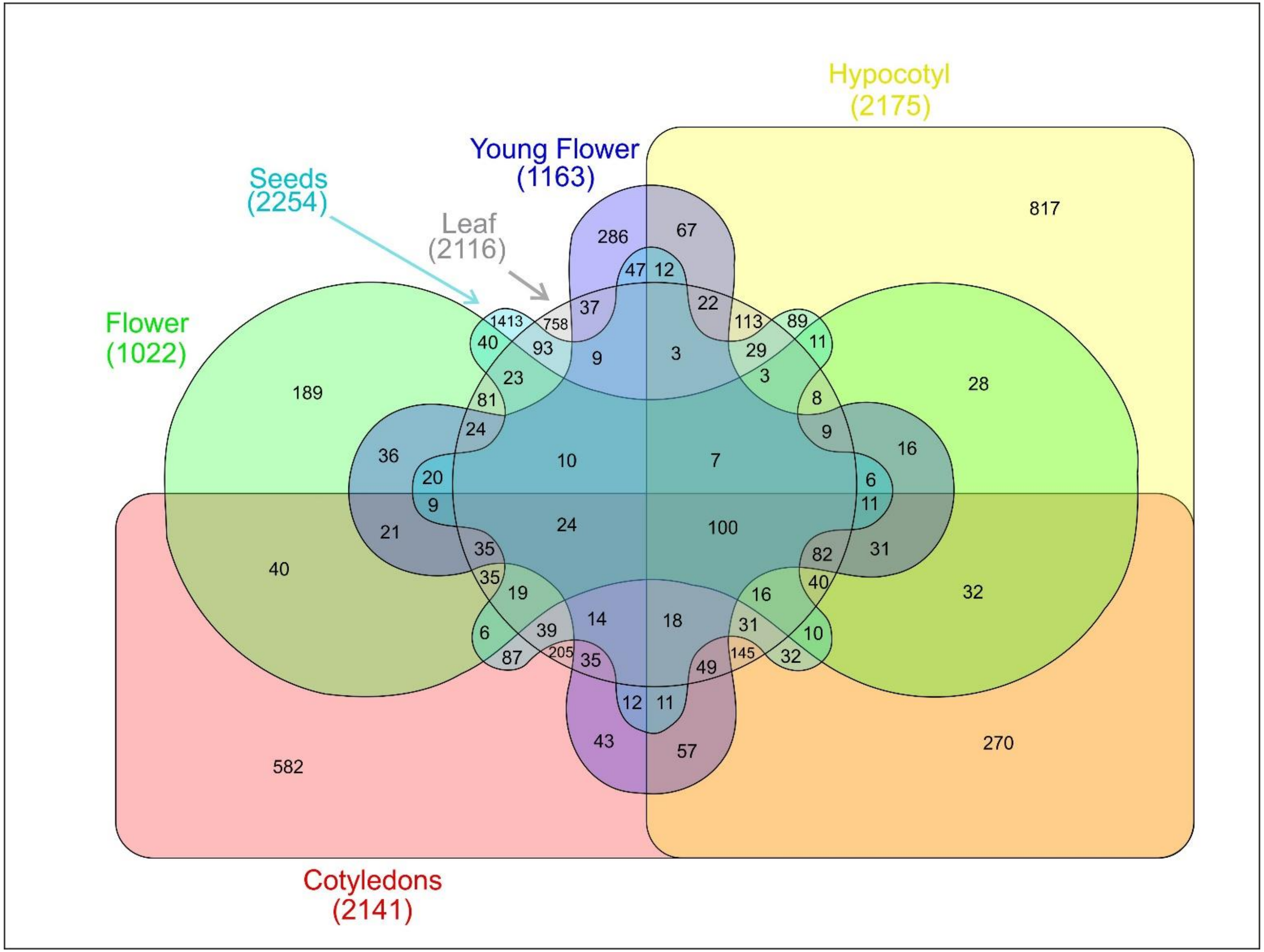

Venn diagram of genes that are DE in different sets of organs at 3 hours of cold treatment

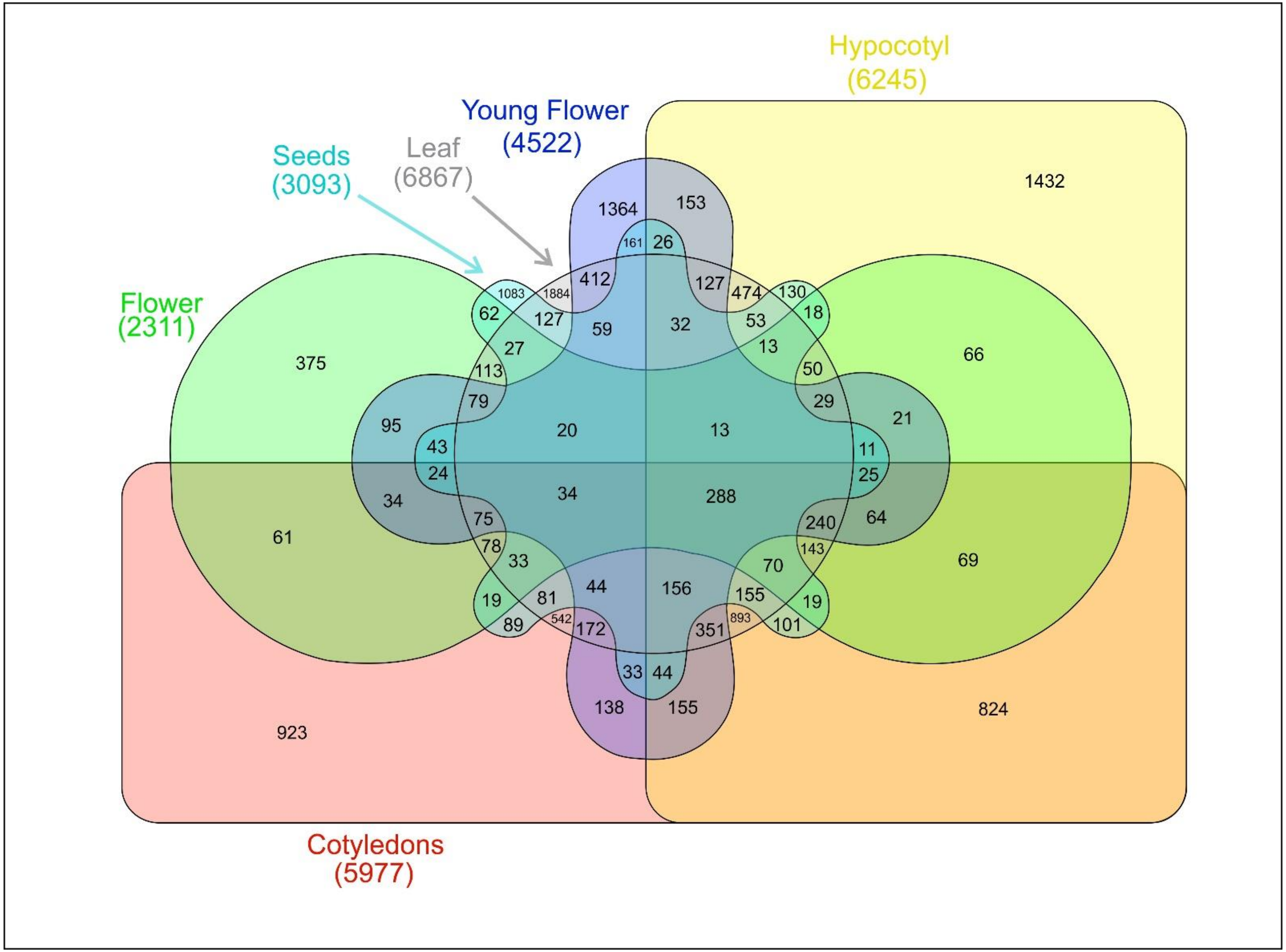

Venn diagram of genes that are DE in different sets of organs at 27 hours of cold treatment
