# Supplementary material for: An update to database TraVA: organ-specific cold stress response in Arabidopsis thaliana
Source: BMC Plant Biol. 2019 Feb 15;19(Suppl 1):49. doi: 10.1186/s12870-019-1636-y (PMC6393959; doi:10.1186/s12870-019-1636-y)
Supplement: Supplementary file 4 — Histogram of fold enrichment in groups of GO terms enriched in common for all or at least five samples genes (PDF 7 kb) [file 12870_2019_1636_MOESM4_ESM.pdf]

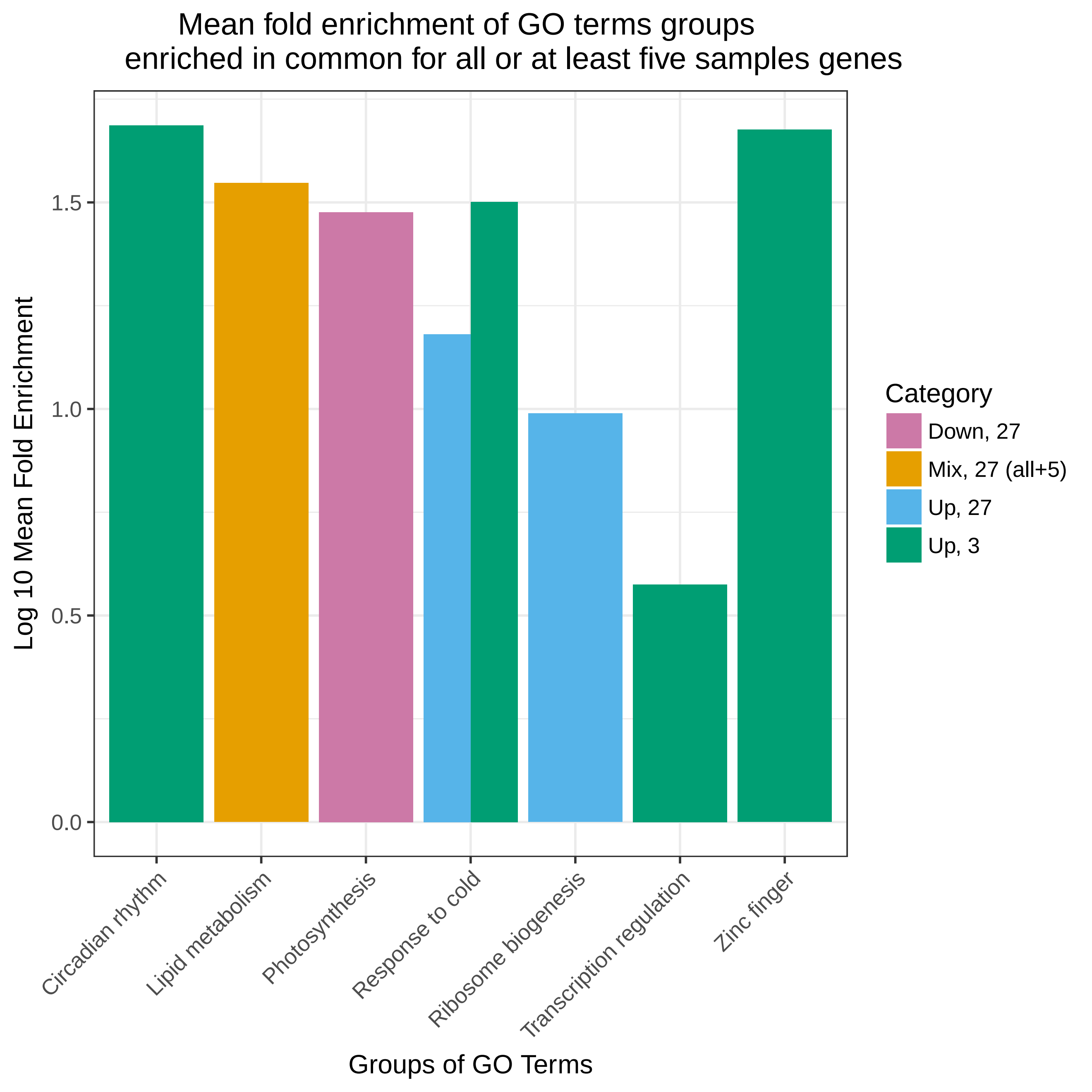

Mean fold enrichment of GO terms groups
enriched in common for all or at least five samples genes
Log 10 Mean Fold Enrichment
0.0
0.5
1.0
1.5
Circadian rhythm
Lipid metabolism
Photosynthesis
Response to cold
Ribosome biogenesis
Transcription regulation
Zinc finger
Groups of GO Terms
Category
Down, 27
Mix, 27 (all+5)
Up, 27
Up, 3
